# A review of chest radiographic patterns in mild to moderate novel corona virus disease 2019 at an urban hospital in Ghana

**DOI:** 10.4314/gmj.v54i4s.8

**Published:** 2020-12

**Authors:** Kwame Asare-Boateng, Yaw B Mensah, Naa Adjeley Mensah, Joseph Oliver-Commey, Ebenezer Oduro-Mensah

**Affiliations:** 1 Radiology Department, Ga East Municipal Hospital, Accra, Ghana; 2 Department of Radiology, University of Ghana Medical School, Korle Bu, Accra; 3 University of Ghana, Regional Institute of Population Studies, Legon, Accra; LEKMA Hospital, Accra, Ghana

**Keywords:** COVID-19, Ghana, Chest radiograph, RT-PCR

## Abstract

**Introduction:**

The novel corona virus disease 2019 (COVID-19) was diagnosed in Wuhan, China in December 2019 and, in Ghana, in March 2020. As of 30^th^ July 2020, Ghana had recorded 35,142 cases. COVID-19 which can be transmitted by both symptomatic and asymptomatic individuals usually manifest as pneumonia with symptoms like fever, cough, dyspnoea and fatigue. The current non-availability of a vaccine or drug for COVID-19 management calls for early detection and isolation of affected individuals. Chest imaging has become an integral part of patient management with chest radiography serving as a primary imaging modality in many centres.

**Methods:**

The study was a retrospective study conducted at Ga East Municipal Hospital (GEMH). Chest radiographs of patients with mild to moderate disease managed at GEMH were evaluated. The age, gender, symptom status, comorbidities and chest x-ray findings of the patients were documented.

**Results:**

11.4 % of the patients had some form of respiratory abnormality on chest radiography with 88.9% showing COVID-19 pneumonia features. 93.8% showed ground glass opacities (GGO), with 3.1% each showing consolidation (CN) only and CN with GGO. There was a significant association between COVID-19 radiographic features and patient's age, symptom status and comorbidities but not with gender.

**Conclusion:**

Most radiographs were normal with only 11% showing COVID-19-like abnormality. There was a significant association between age, symptom status and comorbidities with the presence of COVID-19 like features but not for gender. There was no association between the extent of the lung changes and patient characteristics.

**Funding:**

None declared

## Introduction

The novel corona virus disease 2019 (COVID-19) was first diagnosed in Wuhan, China in December 2019^1^–^4^ and named Corona Virus Disease-2019 by the World Health Organization (WHO) on February 13, 2020.^5^ Ghana recorded its first two cases on 12^th^ March 2020 both of which were imported.^6^ As at 30^th^ July, 2020, Ghana had recorded 35,142 cases with 31,286 recoveries and 175 deaths.^7^

COVID-19 patients usually have a pneumonia and show symptoms like fever, cough, dyspnoea and fatigue and less commonly rhinorrhoea, sore throat, loss of smell, loss of taste and diarrhoea.^8^–^10^ The disease can be transmitted by both symptomatic and asymptomatic individuals.^8^,^11^

There is currently no widely accepted vaccine or drug for treatment of corona virus disease 2019. Early detection and isolation of affected individuals from the general population is key in preventing the spread of the disease.^12^

The gold standard for diagnosing COVID-19 is viral nucleic acid assay test by reverse transcription polymerase chain reaction (RT-PCR). This method of diagnosis has a high sensitivity but fraught with challenges like long processing time, relative unavailability, sampling errors and insufficient stability.^12^ Chest imaging has become an integral part of the management of COVID-19 pneumonia.

In several centres, chest radiography is seen as a primary imaging modality for patient management.^1^ Its acceptance has been potentiated by the long turn-around time of RT-PCR tests and the fact that it is easier to decontaminate compared to computed tomography (CT) scan which is seen as a more sensitive modality.^8^ The American College of Radiology (ACR) therefore recommends the use of portable chest radiography in the management of COVID-19 patients to facilitate easy decontamination of the equipment.^2^,^8^

Chest radiography has a low sensitivity (69%) compared to CT scan (97%) and RT-PCR (91%) in diagnosing COVID-19 but has the potential of predicting disease outcome in the current pandemic.^1^ Reports on the nature and distribution of abnormalities on chest radiography in COVID-19 patients are varied. Interpretation of chest xray (CXR) findings may be confounded by underlying comorbid conditions.^12^

A multinational consensus statement from the Fleischner Society reported in the July 2020 edition of the CHEST journal made recommendations on the use of chest imaging in COVID-19 disease based on three severity scenarios. Chest imaging should only be used in mild disease when the patient is at a high risk of disease progression. Chest imaging was recommended for moderate to severe and severe disease especially in situations with worsening respiratory status. The group of experts further suggested that in resource constrained communities where CT scan is not readily accessible chest x-ray could be used except in situations where there is severe respiratory compromise.^13^

The most common findings on chest radiography are lung consolidation (CN) and ground glass opacity(GGO).^2^,^14^ These changes tend to be in multiple lobes compared to changes in community acquired bacterial pneumonia which tend to involve one lobe on one side of the lung. They tend to be bilateral and have lower zone distribution but pleural effusion is rare.^2^,^8^,^10^

A lot of studies have been done on imaging patterns and role in the management of COVID-19 during this pandemic. No large study has been done on some of these findings which will build local capacity in the management of the condition. This study was designed to document the radiographic features in patients with mild and moderate COVID-19 disease and also find out if there is a relationship between the radiographic findings and patient characteristics. The study also sought to find out if chest radiography had a role to play in the management of mild to moderate COVID-19 patients.

## Methods

The study is a retrospective study conducted at Ga East Municipal Hospital (GEMH). Ethical clearance was obtained from the Ghana Health Service Ethics Review Committee (GHS-ERC 006/05/20). Codes, rather than personal identifiers were used throughout the process of data collection and analysis to ensure anonymity and maintain patient confidentiality.

The chest radiographs used in this study were images of patients with mild to moderate disease managed at the Ga East Municipal Hospital between 11^th^ April, 2020 and 17^th^ July, 2020. Ga East Municipal Hospital is a modern state of the art 100 bed-capacity hospital located in the Ga East Municipality in the Greater Accra Region of Ghana. It serves communities on the eastern side of Accra and Aburi in the Eastern Region of Ghana. This facility has since the onset of COVID-19 been used as a treatment centre for patients with mild and moderate disease.

Chest radiographs of patients were acquired using a Philips Duradiagnost F30 digital radiography equipment. For most of the patients, the images were taken in posteroanterior projections with an object image distance of 1.8m and at the end of inspiration. Twenty patients could not stand for their radiograph to be taken hence had their images taken in the anteroposterior projections. The images were reviewed by two general radiologists with between 8 and 11 years of experience and consensus was reached on the findings before they were documented.

The age, gender, symptom status, co-morbidities and chest x-ray findings of the patients were also documented. Chest x-ray findings were documented based on a method adopted by Wong et al.^8^ The pattern of opacification was grouped into consolidation, ground glass opacity, pulmonary nodules and other (if finding did not fit any of these patterns). The disease distribution was recorded as peripheral dominance, perihilar dominance or neither as well as in the upper, middle or lower zones of the lung. The site of the lung abnormality was noted as right, left or bilateral. The presence of pleural effusion and non-respiratory findings were also noted. The extent of the disease was quantified by assigning a score of 0–4 to each lung depending on the extent of involvement by consolidation or GGO (0 = no involvement; 1 = <25%; 2 = 25–50%; 3 = 51–75%; 4 = >75% involvement). The age, gender, symptom status, presence or absence of the comorbidities as well as the imaging findings were summarised using tables and charts.

Tests of association, Pearson's R and Pearson Chi-Square were used to find relationships between features suggestive of COVID-19 and age, gender, symptom status and comorbidities as well as between the lung finding score and age, gender, symptom status and comorbidities. The radiologists were blinded to symptom status and comorbid conditions during the evaluation.

## Results

Chest radiographs of 334 patients were evaluated out of which 194 (58.1%) were that of males and 140 (41.9%) were that of females. The mean age of the patients was 40.2 years with a standard deviation of 14.8 years. The 30–39-year age-group had the highest number of patients, 99 (29.6%) followed by 20–29-year age-group 64(19.2%) and 40–49-year age-group, 62 (18.6%) as shown on Table 1.

**Table 1:** Age distribution of study patients

Age-group	Female N(%)	Male N(%)	Total N(%)
**<19**	14(10)	6(3.1)	20(6)
**20–29**	29(20.7)	35(18.0)	64(19.2)
**30–39**	38(27.1)	61(31.4)	99(29.6)
**40–49**	23(16.4)	39(20.1)	62(18.6)
**50–59**	16 (11.4)	34(17.5)	50(15)
**60–69**	16(11.4)	12(6.1)	28(8.4)
**>70**	4(2.9)	7(3.6)	11(3.3)
**Total**	140(100)	194(100)	334(100)

Two hundred and fifty-four patients (76.0%) were asymptomatic and 63 (18.9%) were symptomatic. Seventeen patients (10.2%) did not have their symptoms documented. Eighty-one patients out of the 334 patients had documented comorbid conditions out of which 38 (46.9%) had hypertension only, 10 (12.3%) had hypertension and diabetes mellitus and 10 (12.3%) had only diabetes as shown on Table 2. The comorbidities of the remaining 23 patients have also been documented on Table 2.

**Table 2:** Comorbid conditions of study patients

Condition	N(%)
**Asthma**	4(4.9)
**Asthma & Hypertension**	3(3.7)
**Benign Breast Mass**	1(1.2)
**Congestive Cardiac Failure**	1(1.2)
**Congestive Cardiac Failure & Hypertension**	1(1.2)
**Diabetes Mellitus**	10(12.3)
**Goitre**	1(1.2)
**Hypertension**	38(46.9)
**Hypertension & Breast Cancer**	1(1.2)
**Hypertension and Diabetes Mellitus**	10(12.3)
**Hypertension and Peptic Ulcer Disease**	1(1.2)
**Peptic Ulcer Disease & Hepatitis B**	1(1.2)
**Peptic Ulcer Disease**	6(7.4)
**HIV/AIDS**	1(1.2)
**Sickle Cell Disease**	2(2.5)

**Figure 1:**
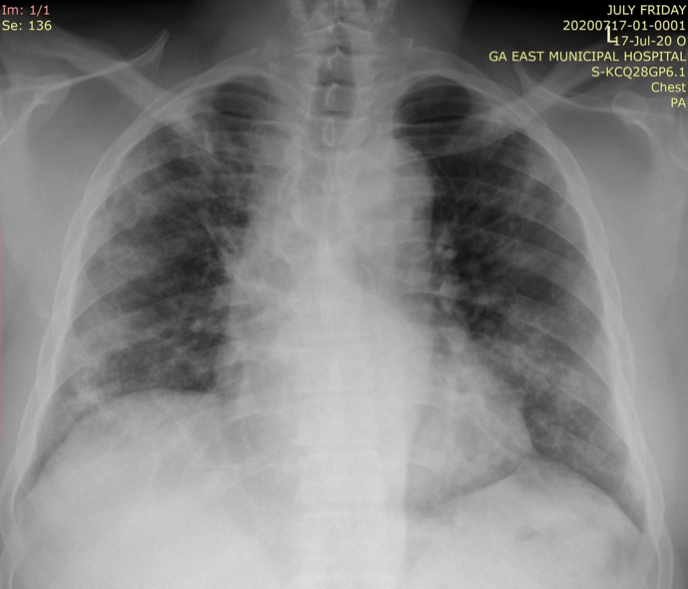
Posteroanterior chest radiograph showing bilateral ground glass opacities in all the zones of both lung fields - Moderate to Severe Disease. Also noted are elevated right hemidiaphragm, tenting of the left hemidiaphragm and unfolding of the aorta

**Figure 2:**
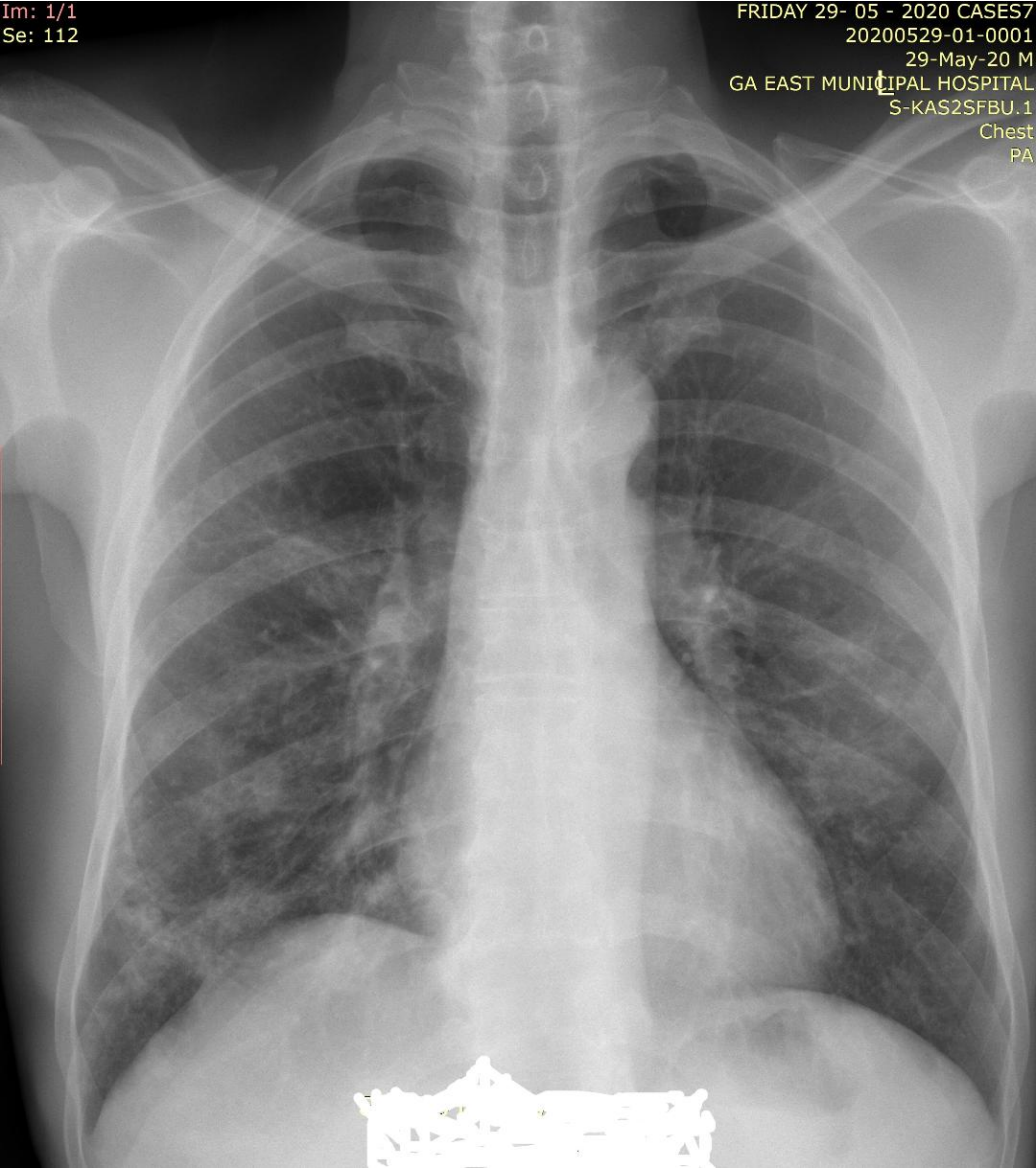
Posteroanterior chest radiograph showing patchy ground glass opacities in the right lung base and subtle ground glass opacities in both middle zones- Mild Disease

Thirty-six (11.4 %) of the 334 patients had some form of respiratory abnormality on their chest radiograph; thirtytwo (88.9%) showed features consistent with COVID-19 pneumonia while four (11.1%) showed other features including three with Koch's disease (8.3%) and one with solitary pulmonary nodule (2.8%). For the 32 who had features consistent with COVID-19 pneumonia, 30 of them (93.8%) showed GGO (Figures 1&2), with 1 patient (3.1%) each showing CN only and CN with GGO.

With respect to the distribution of the COVID-19-like features on chest radiograph, 18 (56.3%) of them showed lesions in both perihilar and peripheral zones, with no dominance in each of the zones. In 13 patients (40.6%), the lesions showed peripheral dominance with one patient (3.1%) showing perihilar dominance. Twenty-three (71.9%) of the patients showed bilateral distribution of the lesions with 8 patients (25%) showing lesions only on the right and one patient (3.1%) showing lesions only on the left. Eleven lesions, (34.4%), were noted in the lower zone only, followed by 10 (31.3%) in all the three zones, 8 (25%) in the middle and lower zones, and 3 (9.4%) in the middle zone. There was no lesion that was found only in the upper zone.

The extent of lung disease score for the patients with radiographic features consistent with COVID-19 pneumonia was also documented. Nine patients (28.1%) had a score of 1; 5 patients (15.6%) had a score of 2; another 5 patients (15.6%) manifested a score of 8; 4 patients (12.5%) had a score of 6; while 3 patients (9.4%) had a score of 5. Each category of radiographs with scores of 3, 4 and 7 was noted in 2 patients (6.25%).

Thirty-five patients (10.5%) had non-respiratory abnormalities. Of these 34 (97.1%) had cardiomegaly and one patient (2.9%) had an anterior mediastinal lesion. Of those who had cardiomegaly, 11 patients (32.4%) were in their sixth decade; 8 patients (23.5%) were in their fourth decade; 6 patients (17.6%) were in their fifth decade; another 6 patients (17.6%) were in their seventh decade and 3 patients (8.8%) were in their third decade. None of the patients was less than 20 years of age. The radiographs of 10 females (7.2% of the total female population) and 22 males (11.4% of the total male population) showed features suggestive of COVID-19 pneumonia.

With respect to their symptom status, 18 asymptomatic patients (7.1% of the total asymptomatic patients) showed COVID-19 like radiographic features while 14 symptomatic patients (22.2% of the total symptomatic patients) showed same. The age distribution and comorbidities for those with COVID-19-like radiographic features have been shown on Tables 3 and 4.

**Table 3:** Proportion of patients with COVID-19 radiographic features

Age-group	No. of Patients with Abnormality	Total Number of Patients	Percentage of Patients with Abnormality
**<19**	0	20	0
**20–29**	2	64	3.1
**30–39**	8	99	8.1
**40–49**	8	62	12.9
**50–59**	7	50	14
**60–69**	7	28	25
**>70**	2	11	18.2
**Total**	34	334	

**Table 4:** Comorbidities of patients with COVID-19-like abnormalities

Condition	No. with Abnormality	No. of Patients	% with Abnormality
**Asthma**	0	4	0
**Asthma & Hypertension**	1	3	33.3
**Benign Breast Mass**	0	1	0
**Congestive Cardiac Failure**	0	1	0
**Congestive Cardiac Failure & Hypertension**	0	1	0
**Diabetes Mellitus**	5	10	50
**Goitre**	0	1	0
**Hypertension**	3	38	7.9
**Hypertension & Breast Cancer**	1	1	100
**Hypertension and Diabetes Mellitus**	6	10	60%
**Hypertension and Peptic Ulcer** **Disease**	0	1	0
**Peptic Ulcer Disease & Hepatitis B**	0	1	0
**Peptic Ulcer Disease**	0	6	0
**HIV/AIDS**	0	1	0
**Sickle Cell Disease**	1	2	50%
**No comorbidity**	5	64	7.8
**Not stated**	12	189	6.3
**Total**	34	334	

There was a significant association between the presence of radiographic features consistent COVID-19 and patient's age, presence of symptoms and comorbidities but not with gender. However, there was no association between the age, gender, symptom status and comorbidities and the extent of the lung changes. These are shown on Tables 5 and 6.

**Table 5:** Test of association between presence of COVD-19 features and Patient characteristics

Patient Characteristics	Test Statistic	p-Value
**Age**	Pearson's R	<0.001
**Gender**	Pearson Chi-Square	0.462
**Symptom Status**	Pearson Chi-Square	<0.001
**Comorbidities**	Pearson Chi-Square	<0.001

**Table 6:** Test of Association between score of extent of lung disease in patients with COVD-19 features and patient characteristics

Patient Characteristics	Test Statistic	p-Value
**Age**	Pearson's R	0.055
**Gender**	Pearson Chi-Square	0.701
**Symptom Status**	Pearson Chi-Square	0.473
**Comorbidities**	Pearson Chi-Square	0.108

## Discussion

There were more males (58.1%) than females (41.9%) in this study which was in agreement to what was found by Wu et al and Zhou et al who reported 60% and 54% for males and 40% and 46% for females respectively.^15^,^16^ Wong et al and Ai et al reported more females than males. This seems to suggest that the relative proportions of male to female with COVID-19 depends on the relative proportion of male and females at the location where the study was done and not on the gender per se. 8,12

The mean age of the study population was 40.2 years. This was close to 42.9 years reported by Wu et al, but lower than that reported by Wong et al, Zhou et al and Ai et al who reported 56 years, 51 years and 52.4 years respectively.^12^,^15^–^17^The younger generation tend to have milder disease, it is therefore not surprising that conducting a study at a centre which sees only mild and moderate cases will have a lower mean age.^1^ This belief is further supported by the fact that about two-thirds (67.4%) of the patients in this study were between 20 and 49 years of age.

This study also found out that there were more than 76% asymptomatic patients among the study population. This could be explained by the age characteristics of patients enrolled in the study. The finding will also support the notion that asymptomatic patients may be infecting people more than symptomatic patients.

Close to 25% of the patients had comorbid conditions. Hypertension was the commonest among the patients, with 61.6% of them having hypertension either alone or with other conditions. This was followed by diabetes mellitus 24.6% either alone or with hypertension and Asthma as the third 8.6%. These findings compare favourably with what was reported by Toussie et al, in their study to find out the outcome of COVID-19 pneumonia in young adults and middle age individuals.^1^

Only 11.4% of the patients showed radiographic features suggestive of COVID-19 pneumonia. This seems to support the suggestion by the Fleishner Society to limit radiography to moderate to severe and severe disease.^13^ Similar statements have been made by ACR and Society of Thoracic Radiologists.^1^ These prominent societies have also spoken against screening populations for COVID-19 with chest radiography. Again, the low yield in this study seems to confirm that notion as well.

Ground glass opacities and consolidation were the two most common features noted on chest radiography. This is consistent with what has been reported by many authorities.^1^,^8^,^12^,^16^ This study found more GGO far in excess of consolidation which was at variance with what was found in other chest radiography studies where consolidation was the more common finding.^8^ The explanation those authors gave was that it was easier to see consolidation on chest radiography than ground glass opacities. Indeed, studies that used CT scan tend to confirm that there are more patients with GGO on their chest images than consolidation.^15^–^17^

Most (56.3%) of the lesions had both peripheral and perihilar distribution, 40.6% showed peripheral distribution and 72% of the lesions were bilateral. None of the lesions showed absolute upper zone distribution. The lesions were mostly in the middle and lower zones with about a third in all the three zones. These findings have been corroborated by almost all researchers who have reported on COVID-19 pneumonia.^2^,^8^,^12^,^15^–^17^

Consistent with the type of patient population, most (90.4%) of the patients did not have any radiographic features of COVID-19 pneumonia. Of those with some COVID-19 pneumonia findings, majority (43.7%) had mild disease with scores of 1 and 2. Those with moderate disease, scores of 3 and 4 were about 12.5%, with the rest having varying degrees of severe disease. This finding was in line with Wong et al^8^ who also found most of their patients having mild disease. This supports the view that though COVID-19 is highly infectious, majority of the patients will have mild disease. Again, this may explain why over 70% of patients were asymptomatic.^8^

Ten percent of the patients had cardiomegaly. Fifty percent of these were between their third and fifth decades. This points out a possible additional benefit of radiography in that it may also help identify patients with significant non respiratory system findings such as cardiomegaly and anterior mediastinal lesions as noted in this study.

There was a significant association between the presence of radiographic features consistent with COVID-19 and patient's age, presence of symptoms and comorbidities but not with gender. The study by Toussie et al^1^ showed that, like this study, there was a significant association between age and the presence of some comorbidities with abnormal COVID-19-like finding and severity of these findings on chest x-ray.^1^

While they found gender had an association with the presence of abnormal COVID-19-like findings, our study did not show that association. In addition, Toussie et al^1^ found an association between age, gender and some comorbid conditions like HIV and obesity and the severity of the chest radiography findings, our study did not find that. However both studies did not find any association between severity of lung findings and conditions like hypertension and diabetes which formed the majority of the comorbidities of our patients.^1^

Being a retrospective study, we encountered some challenges as some patients' symptoms and possible comorbidities were not accurately documented and it was difficult to obtain such information after the discharge of the patients. Again, none of them had follow up radiographs after the initial radiograph, thus could not document the progress of the disease.

## Conclusion

The study showed that most of the patients did not have any significant finding on chest radiograph with only about 11% showing significant abnormality. Among those with abnormalities there was a significant association between their age, symptom status and comorbidities with the radiographic findings but no such association was found with gender. There was also no association between the extent of the lung changes and patient characteristics. This study therefore supports the position of the Fleischner Society's stance on the use of chest radiograph in mild disease.
